# Impact of the Amyotrophic Lateral Sclerosis Disease on the Biomechanical Properties and Oxidative Stress Metabolism of the Lung Tissue Correlated With the Human Mutant SOD1^G93A^ Protein Accumulation

**DOI:** 10.3389/fbioe.2022.810243

**Published:** 2022-02-25

**Authors:** Duygu Aydemir, Anjum Naeem Malik, Ibrahim Kulac, Ayse Nazli Basak, Ismail Lazoglu, Nuriye Nuray Ulusu

**Affiliations:** ^1^ Department of Medical Biochemistry, School of Medicine, Koc University, Istanbul, Turkey; ^2^ Koc University Research Center for Translational Medicine (KUTTAM), Istanbul, Turkey; ^3^ Manufacturing and Automation Research Center, Department of Mechanical Engineering, Koc University, Istanbul, Turkey; ^4^ Department of Pathology, Koc University School of Medicine, Istanbul, Turkey; ^5^ Suna and İnan Kirac Foundation, Neurodegeneration Research Laboratory, NDAL-KUTTAM, School of Medicine, Koc University, Istanbul, Turkey

**Keywords:** ALS, biomechanical test, stiffness, oxidative stress, SOD1

## Abstract

Amyotrophic lateral sclerosis (ALS) is the most common motor neuron disease, and ALS incidence is increasing worldwide. Patients with ALS have respiratory failure at the disease’s end stages, leading to death; thus, the lung is one of the most affected organs during disease progression. Tissue stiffness increases in various lung diseases because of impaired extracellular matrix (ECM) homeostasis leading to tissue damage and dysfunction at the end. According to the literature, oxidative stress is the major contributor to ECM dysregulation, and mutant protein accumulation in ALS have been reported as causative to tissue damage and oxidative stress. In this study, we used SOD1^G93A^ and SOD1^WT^ rats and measured lung stiffness of rats by using a custom-built stretcher, where H&E staining is used to evaluate histopathological changes in the lung tissue. Oxidative stress status of lung tissues was assessed by measuring glucose 6-phosphate dehydrogenase (G6PD), 6-phosphogluconate dehydrogenase (6-PGD), glutathione reductase (GR), glutathione s-transferase (GST), catalase (CAT), and superoxide dismutase 1 (SOD1) levels. Western blot experiments were performed to evaluate the accumulation of the SOD1^G93A^ mutated protein. As a result, increased lung stiffness, decreased antioxidant status, elevated levels of oxidative stress, impaired mineral and trace element homeostasis, and mutated SOD1^G93A^ protein accumulation have been found in the mutated rats even at the earlier stages, which can be possible causative of increased lung stiffness and tissue damage in ALS. Since lung damage has altered at the very early stages, possible therapeutic approaches can be used to treat ALS or improve the life quality of patients with ALS.

## Introduction

Amyotrophic lateral sclerosis (ALS) is the most common and severe motor neuron disease affecting upper and lower motor neurons in the brain, brain stem, and spinal cord. ALS is categorized as sporadic ALS (sALS) with no known cause and familial ALS (sALS) resulting from the genetic inheritance (Aydemir and Ulusu, 2020b; Burlando et al., 2020). Several genes have been identified as causative or disease-modifying fALS and sALS, such as C9ORF72, SOD1, TARDBP, and FUS. D1 is the most studied gene mutation in ALS disease and one of the essential antioxidant enzymes involved in redox sensing, oxidative stress metabolism, and signal transduction (Jawaid et al., 2010; Gill et al., 2019; Cassina et al., 2021).

The lung is one of the most affected organs during ALS progression, and patients with ALS have respiratory failure at the end stages of the disease leading to death (Aydemir and Ulusu, 2020b). Therefore, handling respiratory failure aims to prolong survival and improve quality of life during ALS progression (Niedermeyer et al., 2019). Lung tissue consists of elastin, collagen, and proteoglycans representing the connective tissue, giving specific mechanical properties to the lung. Biomechanical properties of the lung tissue such as stiffness, force, or elastic modulus impaired in various diseases including Parkinson’s Disease, pulmonary fibrosis, cancer, asthma, emphysema, chronic obstructive pulmonary disease (COPD), and pulmonary arterial hypertension (PAH) disease; however, biomechanical changes associated with the histopathological alterations of the lung tissue have not been studied in the ALS disease (Mariappan et al., 2011; Burgstaller et al., 2017). Elastic recoil and mechanical stability are essential for healthy lung function regulated by the biochemical and biomechanical signals (Hynes, 2009). However, aging leads to impaired lung function, such as tissue stiffness, because of alterations in the biomechanical and biochemical properties of the lung tissue (Sicard et al., 2018; Racca et al., 2020).

Tissue stiffness regulates tissue function, homeostasis, and cellular activity, thus decreased or increased tissue stiffness is associated with dysregulated cell behaviors and disease progressions such as pulmonary fibrosis, liver cirrhosis, ALS, and scleroderma (Discher et al., 2009; Liu et al., 2015; Guimarães et al., 2020). Lung tissue stiffening can result from several mechanisms, including extracellular matrix (ECM) dysregulation, protein aggregation, aging, impaired mineral metabolism, and elevated levels of trace elements (White, 2015). ECM metabolism and homeostasis are affected by the tissue’s redox status, and elevated levels of oxidative stress are tightly associated with ECM dysregulation (Watson et al., 2016). Since the SOD1 enzyme is one of the major antioxidant enzymes, a mutation in this gene causes impaired oxidative stress metabolism and tissue damage (Hayashi et al., 2016; Gill et al., 2019; Martínez-Palma et al., 2019).

Biomechanical and biochemical aspects of the lung are affected by the oxidative stress status of tissue (Brent et al., 2020); thus, we evaluated histopathological, biomechanical, and biochemical changes of the lung tissues of SOD1 G93A mutated rats (SOD1^G93A^) male rats comparing with the wild type rats (SOD1^WT^) by eliminating the effects of the aging first time in the literature. We hypothesized that SOD1^G93A^ mutation causes biomechanical impairment in the lung tissue due to elevated oxidative stress. Our study may help to explain possible mechanisms behind the lung dysfunction in the ALS disease that can be used as potential therapeutic approaches in the future to improve the life quality and survival time of patients with ALS.

## Materials and Methods

### Animal Studies

Six male and six female hemizygous SOD1^G93A^ mutated albino rats were purchased from Taconic with catalog number NTac:SD-Tg(SOD1G93A) L26H. Animals were inbred at the Animal Research Facility of Koc University, and 70 male rats weighing 140–650 g were used during the experiments. Power analysis was performed to determine the number of animals used in the study. Animals were housed in the polycarbonate cages with stainless steel covers in an air-conditioned room (12 h light/dark cycle with a temperature of 22 ± 2°C and relative humidity of 50 ± 5%). Animals had free access to tap water and standard rat pellet food. The Ethics Committee approved all experimental procedures and animal use of Koc University with the number 2019.HADYEK.006.

Animals were followed every week by weighing and controlling their movements, indicating disease progression. First, we have divided our animals into ten groups based on age as 0 (40–45 days old), A (70–75 days old), B (90–95 days old), C (110–115 days old), and D (130–135 days old), and each group is divided into two subgroups according to their mutation status respectively as SOD1^WT^ and SOD1^G93A^. Group C accounts for the early stage, and group D refers to the late stage of the ALS disease. We have started to observe disease symptoms in group C, and therefore we have indicated group C as early-stage and group D as the late stage. Animals were anesthetized under isoflurane, and then blood was taken from the heart of the rats. Animals were sacrificed under anesthesia via guillotine to minimize the pain and suffering of animals. All tissues were removed and washed with ice-cold sterile saline solution to wash out blood following sacrification. Half of the lung tissue was frozen in the dry ice and stored at −80°C, where the rest of the lung was placed in the 10% formalin to perform histopathological examinations.

### Genotyping of SOD1^WT^ and SOD1^G93A^ Rats

25 mg of rat tail tissue was used to isolate DNA via DNeasy Blood & Tissue Kit (QIAGEN, Germany). 20 ng/ul of DNA was used to perform PCR with forward and reverse primers (Forward primer: 5′ GTG GCA TCA GCC CTA ATC CA 3′ and Reverse primer: 5′ CAC CAG TGT GCG GCC AAT GA 3′). Cycling conditions were denaturation at 95°C for 1 min, extension at 95°C 15s, annealing 62.1°C 15s and then at 72°C 20s, final extension 72°C for 8 min 34 cycles. After PCR was done, products were loaded into the 2% agarose gel to evaluate the corresponding bands. NDAL Laboratory conducted genotyping of SOD1 rats at the Koc University Hospital.

### Experimental Setup of the Biomechanical Tests

**FIGURE 1:**
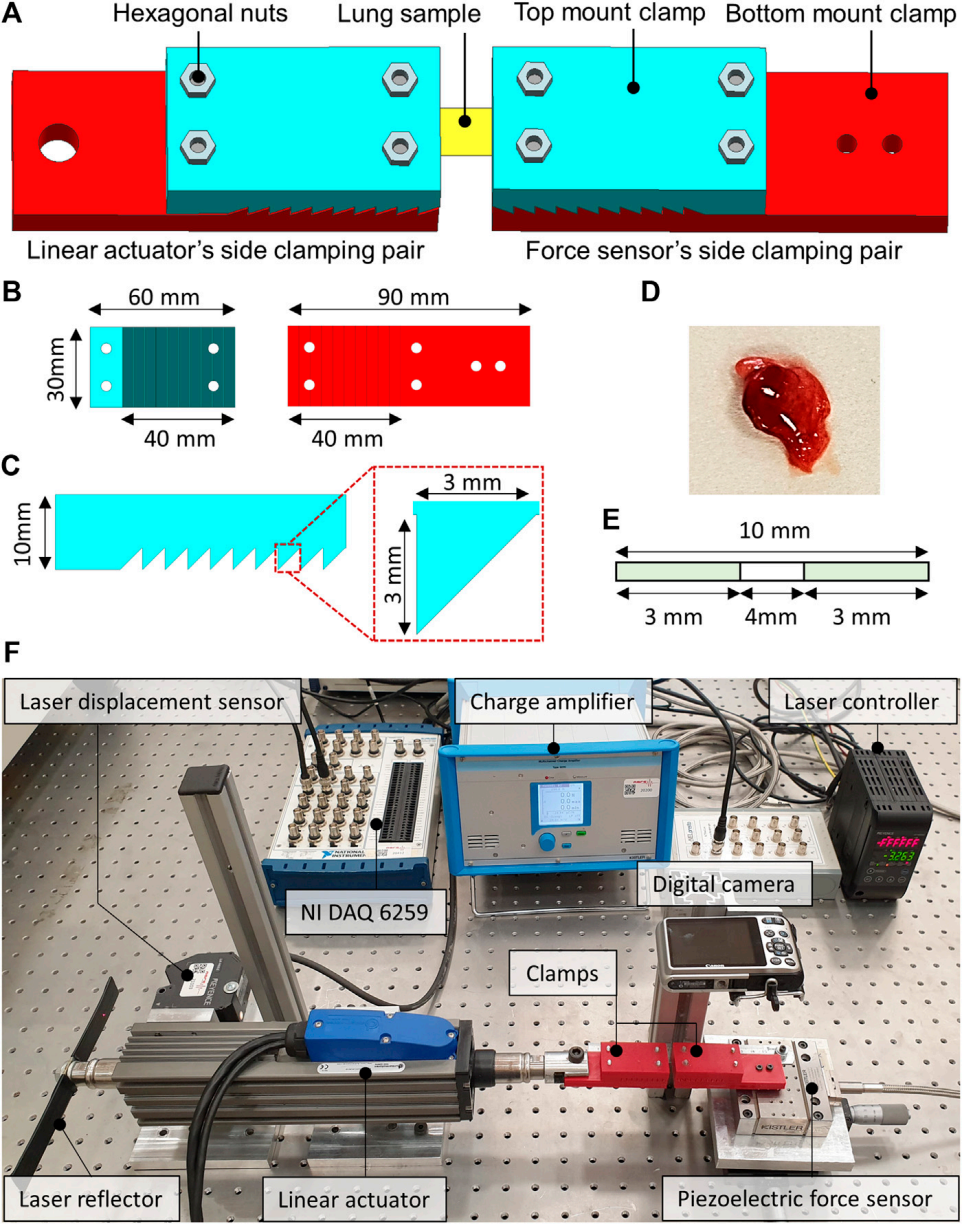
The computer-aided design (CAD) of the sample holding clamps and the dissected lung sample of the rat. **(A)** The locking of the lung sample between the force sensor side and the linear actuator side clamps. **(B)** CAD model of the top and bottom mount of the clamps. **(C)** Zoomed view of the saw-tooth structure on the clamps along with the dimensions. **(D)** Dissected lung sample of the rat. **(E)** The schematics illustrate the dimensions of the studied lung sample. **(F)** The custom-built tensile testing experimental setup indicates the laser displacement sensor, the force sensor, the linear actuator, and the data acquisition system.

Uniaxial tensile testing using a custom-built experimental setup which was comprised of a pre-calibrated piezoelectric-based precise force sensor (Kistler 9256C1), a laser type displacement sensor (Keyence LK-H052), an electromagnetic-based linear actuator (Dunkermotoren STA-25), a pair of additively manufactured tissue holding clamps and a high-resolution digital camera for the subsequent slippage analysis of the lung samples. The force sensor was interfaced with the data acquisition module (NI-DAQ 9235) through a charge amplifier, whereas; the laser displacement sensor was interfaced with the NI-DAQ through a laser controller (Figure 1).

The clamps were developed by first creating a computer-aided design (CAD) model in Siemens NX 12.0 software. The CAD models of the clamps were then processed using slicing software before manufacturing. The additive manufacturing was performed using a 3D printer, employing the fused filament deposition (FFD) technique and the polylactic acid (PLA) type filament. The layer thickness of 100 microns and the brass type nozzle having a tip diameter of 0.4 mm were selected to manufacture clamps. Figure 1 illustrates the CAD model of the clamps from different orientations and the dissected lung sample of a rat. The additively manufactured tissue holding clamps comprised a pair of top and bottom mount structures with a saw-tooth type locking arrangement to keep the lung samples firmly when locked. The clamping area was 10-teeth, each having a height and width equal to 3 mm. The top and the bottom mounts were locked into each other with the help of the hexagonal nuts enabling the reusability of the additively manufactured clamps for testing multiple samples. One pair of clamps was mounted on the force sensor, whereas the second pair of clamps were attached to the moving shaft of the linear actuator, as depicted in Figure 1. The two pairs of clamps were then positioned to be 4 mm apart from each other. The lung sample was fixed between the two clamps, as demonstrated in Figure 1, and the hexagonal nuts were tightened enough to avoid slippage and sample breakage.

### Experimental Protocol of the Biomechanical Tests

The uniaxial tensile testing of all the samples was performed under ambient conditions of the test room. The test room temperature and relative humidity were maintained at 22–25°C and 60%–65% RH, respectively**.** At the start of every experiment, the laser displacement and the force sensor offsets were zeroed through the laser control module and the charge amplifier. The experiment started by linearly displacing the shaft of the linear actuator at a speed of 0.5 mm/s in the direction opposite to that of the force sensor to apply uniaxial tension on the lung sample. The force sensor remained fixed throughout the experiment. The experiment is completed once the full tearing of the lung sample is observed. The tensile force was measured with the help of a precise piezoelectric force sensor. The charge output of the force sensor was converted into voltages through a charge amplifier. The laser displacement sensor measured the axial displacement. The force and the laser sensor data were acquired at a sampling rate of 1,000 samples/seconds and stored in the comma-separated file format for subsequent analysis.

### Hematoxylin and Eosin Staining

Tissues were collected and washed with ice-cold saline buffer and fixed in 10% formalin and processed by an automated system then embedded in paraffin Sakura Tissue Tek VIP Tissue Processor & Tissue Embedding Station, Leica) Paraffin (FFPE—formalin-fixed paraffin-embedded) blocks were prepared and cut by 4 µm thick sections on glass slides for hematoxylin and eosin (H&E) staining. Before staining tissues, sections on the slides were deparaffinized via xylene by heating at 60°C for 20 min and then rehydrated with firstly alcohol (96%) and then PBS (phosphate-buffered saline). Slides were dipped in Mayer’s hematoxylin for 1 min and washed with distilled water. After that, slides were immersed in eosin for 10 s and washed multiple times with ethanol (70%, 96%, and absolute). Slides were dipped in xylene, and mounting was performed using an automated slide mounting system (Tissue-Tek Prisma, Sakura Finetek USA). This system uses only xylene and a special film for mounting and does not need other mounting media.

### Microwave Digestion

Acidic treatment was used to dissolve serum samples of the rats for ICP-MS analysis. A microwave digestion system (Milestone START D) only equipped with the temperature control sensor was used to dissolve serum samples of SOD1^WT^ and SOD1^G93A^ rats. 100 µl of serum sample was dissolved in 10 ml of 65% SUPRAPURE^®^ nitric acid (HNO_3_) (MERCK, Germany) as described previously in detail (Aydemir et al., 2020c).

### ICP-MS

Samples were diluted at a 1/10 ratio after acidic digestion via microwave digestion and used for the ICP-MS analysis. The trace and mineral element levels of the rat serum samples were evaluated using Agilent 7700x ICP-MS (Agilent Technologies Inc., Tokyo, Japan). External calibration solutions were prepared using the Spex Certiprep Multi-element calibration standard (2A). Data analysis was performed *via* MassHunter software (Aydemir et al., 2020c).

### Tissue Preparation and Mitochondria Isolation

Mitochondria isolation was performed according to the modified method described by Clayton and Shadel (Clayton and Shadel, 2014). The buffer solution was prepared with 1 M sucrose and 100 mM Tris-HCl (pH 7.4) in the ultrapure water. pH was set up to 7.4, and protease inhibitor cocktail Roche cOmplete™, EDTA-free (Merck, Germany) was added into the buffer freshly just before homogenization. Tissue samples weighing 40–100 mg were homogenized in this buffer and then centrifuged at 7,000 g for 20 min at 4°C. The pellet containing mitochondria was separated into the clean tubes and stored at 80°C. The supernatant of each sample was collected and centrifuged at 105,000 ×g for 65 min at 4°C, and the supernatant was stored as the cytosolic fraction at 80°C. 200 mM sodium phosphate buffer (pH 7.4) containing protease inhibitor cocktail was added into the mitochondria lysate after thawing and sonicated via Branson W250 at 70% amplitude for three cycles of 4 s each. Samples were centrifuged at 105,000×g for 65 min at 4°C, and the supernatant was stored as the mitochondrial fraction of the lung at 80°C (Aydemir et al., 2019c). Protease inhibitor cocktails were used to prepare mitochondrial and cytosolic fractions to prevent protease activity since many proteases can degrade proteins of interest (Ryan and Henehan, 2017; Dayal et al., 2020).

### Protein Determination

According to the kit instructions, a Pierce BCA Protein Assay kit (Thermo Scientific, USA) was used to evaluate the soluble protein concentration in all samples by using albumin as standard. Samples were pipetted into the 96 well plates and read at 562 nm using Synergy H1, BIOTEK.

### Enzyme-Linked Immunosorbent Assay for Catalase and SOD1

Prepared tissue lysates were used to evaluate cytosolic CAT and SOD1 activity using the Abbkine Rat Catalase ELISA kit (Abbkine, China). Samples were pipetted on the 96-well plate of the ELISA kit and, after incubated with HRP-conjugated antibody wells, were washed and treated with chromogen solutions according to the kit instructions. After the experimental procedure was done, the absorbance of the samples was read at 450 nm.

### Evaluation of Oxidative Stress Enzyme Activities

Glucose 6-phosphate dehydrogenase (G6PD), 6-phosphogluconate dehydrogenase (6-PGD), glutathione reductase (GR), and glutathione s-transferase (GST) enzyme activities were measured in both cytosol and mitochondria fractions of lung samples as described previously (Aydemir et al., 2019a; Aydemir et al., 2019b; Aydemir et al., 2019c).

### Western Blot

Cytosolic and mitochondrial fractions of the lung tissues were used for western blot analysis to evaluate the SOD1^G93A^ protein accumulation after protein quantification. B8H10 antibody (MEDIMABS, Montreal, CANADA) was used to detect human SOD1^G93A^ aggregates in the tissue of interest. After protein quantification as described above, 30–70 μg protein from each sample was diluted with 4x Laemmli buffer (10% SDS, 50% glycerol, 0.3 M Tris-HCl (pH 6.8), 0.05% bromphenol blue in 50 ml Mili-Q water) containing 10% of beta-mercaptoethanol and samples were incubated at 95°C for 2 min in thermoblock. Then samples were separated on 10% SDS-PAGE with 5% stacking gel using PowerPac™ Universal Power Supply at 90 V for ∼2.5 h. After gel electrophoresis, proteins were transferred to the PVDF membrane for an hour at 150 mA with Bio-Rad wet transfer apparatus at 4°C room. Following that, PVDF membranes were blocked with 5% BSA prepared in phosphate-buffered saline/0.1% Tween (PBS-T) for an hour and probed with primary SOD1 antibody (diluted in 1% BSA in PBST to 1:250) at 4°C overnight as described previously (Otahal et al., 2020).

### Statistical Analysis

The statistical significance of the clinical data was evaluated via GraphPad Software, Inc., USA. Mann Whitney test was used to compare the two groups. All data were represented as the mean ± standard deviation (SD).

## Results

### Biomechanical Changes in the Lung Tissue

**FIGURE 2:**
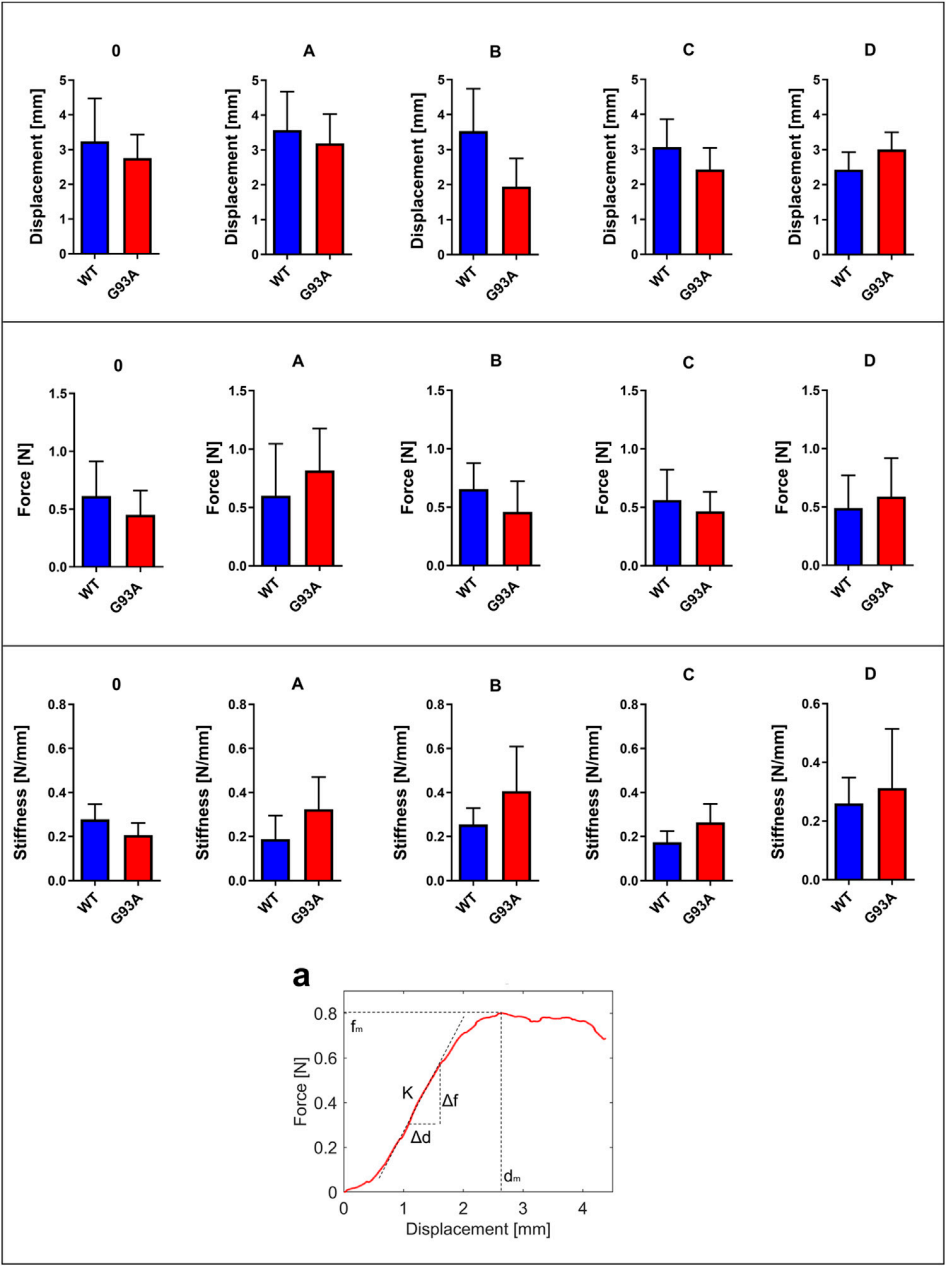
Force, displacement, and stiffness values of lung tissues belonging to SOD1^G93A^ and SOD1^WT^ rats. (a) The stiffness value of lung tissue is calculated as represented by the figure via using displacement and force values. *p ≤ 0.05, **p ≤ 0.001 and ***p ≤ 0.000.

Displacement, force, and stiffness of the SOD1^WT^ and SOD1^G93A^ rats were measured by a custom-built stretcher device represented in Figure 1. Displacement and force have been measured in the lung samples of all rats, and the slope in the linear region of the force versus displacement curve depicts the stiffness of the lung sample (Figure 2) (Wolfla et al., 2010). Lung stiffness increased in the SOD1^G93A^ rats compared to SOD1^WT^ rats in all groups except 0 (Table 1) (Figure 2). Increase in the lung stiffness was not significant in the mutated rats of groups A, B, C and D, however this increase was almost double in the indicated groups in comparison with wild type rats (Table 1).

**TABLE 1:** Lung stiffness of SOD1^G93A^ (SOD1) mutated, and wild-type (WT) rats are represented in the table as mean ± SD.

		Group	Stiffness [N/mm]
MALE	WT	0	0.26 ± 0.06
		A	0.23 ± 0.04
		B	0.23 ± 0.09
		C	0.16 ± 0.13
		D	0.24 ± 0.06
	SOD1	0	0.21 ± 0.19
		A	0.34 ± 0.11
		B	0.47 ± 0.07
		C	0.23 ± 0.09
		D	0.39 ± 0.13

### Histopathological Changes in the Lung Tissue

H&E staining was performed to evaluate histopathological changes in the lung tissues of both SOD1^WT^ and SOD1^G93A^ rats. All tissues were examined after H&E staining, and representative pictures were chosen to demonstrate histopathological changes in the lung. A pathologist performed the histopathological examination and detected the percentage of atelectasis by comparing wild-type and mutated lung samples. No histopathological changes have been observed between SOD1^WT^ and SOD1^G93A^ rats in group 0 (Figure 6); on the other hand, substantial atelectasis (∼30%–60%) and some emphysemas were observed in the SOD1^G93A^ rats of groups A (Figure 4), B (Figure 5), C and D (Figure 6) more common than the SOD1^WT^ group.

### Evaluation of the Trace Element and Mineral Levels in the Lung Tissue

Following acidic digestion, the lung tissue’s trace element and mineral levels were evaluated via ICP-MS. Mg levels slightly decreased in the SOD1^G93A^ rats compared to the SOD1^WT^ rats except for group D; however, this decrease was insignificant (Figure 6). Mg levels increased in the mutated rats in comparison with wild-type rats in groups A, C, and D, and this change was significant in group A (*p ≤ 0.05) (Figure 6). K levels decreased in the mutated rats of groups A (***p ≤ 0.0001), C (***p ≤ 0.0001), and D (**p ≤ 0.001) in comparison with wild-type rats, where K level decreased in the SOD1^G93A^ rats in group 0 (***p ≤ 0.0001) compared to the SOD1^WT^ rats (Figure 6). Fe levels did not show any significant differences between the two groups except in group C, where Fe levels significantly decreased in the mutated rats (**p ≤ 0.001) compared to the wild-type rats (Figure 6). On the other hand, Zn levels reduced in the mutated rats of groups 0, B, C, and D; however, this decrease was significant for group 0 (**p ≤ 0.001) and D (**p ≤ 0.001).

### Human SOD1^G93A^ Mutated Protein Accumulation in the Cytosol and Mitochondria of the Lung Tissues

SOD1^G93A^ mutated protein accumulation (∼16 kDa) in the cytosolic and mitochondrial fractions of the lung tissues belonging to rats was evaluated via B8H10 antibody recognizing mutant human SOD1^G93A^ protein expressed by in the SOD1^G93A^ rat model. Our data showed that human mutant SOD1^G93A^ protein was accumulated in the cytosol and mitochondria of lung tissues belonging to the SOD1^G93A^ rats; however, we did not observe any accumulation in the wild-type rats (Figure 6).

### Evaluation of the Oxidative Stress Metabolism and Pentose Phosphate Pathway Enzymes in the Cytosol and Mitochondria of the Lung Tissues

G6PD enzyme activity decreased in the cytosolic and mitochondrial fractions of lung tissues belonging to the SOD1^G93A^ rats in comparison with SOD1^WT^ rats, indicated decrease was significant in the mitochondria of the groups 0 (*p ≤ 0.05), B (*p ≤ 0.05), C (***p ≤ 0.0001) and D (*p ≤ 0.05), wherein the cytosol for the group B (*p ≤ 0.05) (Figure 7). Same as G6PD, 6-PGD enzyme activity decreased in both cytosolic and mitochondrial fractions of the mutated rats compared to the wild-type rats. For the cytosolic fractions of the lung, 6-PGD enzyme activity significantly reduced in the groups 0 (***p ≤ 0.0001), B (**p ≤ 0.001), and D (**p ≤ 0.001), where this decrease was significant in the mitochondria of the groups C (*p ≤ 0.05) and D (*p ≤ 0.05) (Figure 7).

GR enzyme activity decreased in the mitochondrial fractions of the mutated rats compared to the wild-type rats, and this decrease was significant for group D (***p ≤ 0.0001). For the cytosol, GR activity decreased in the SOD1^G93A^ rats of the groups 0, A, and B; this decrease was significant only for group 0 (*p ≤ 0.05). On the other hand, GR activity slightly increased in the mutated rats belonging to groups C and D (Figure 8). GST enzyme activity increased in the cytosolic fraction of lung samples belonging to the SOD1^G93A^ rats; however, this increase was insignificant in any group. In the mitochondria of lung tissues, GST enzyme activity decreased in the mutated rats (*p ≤ 0.05 for group 0), on the other hand slightly increased in groups B, C, and D (Figure 8).

We further measured SOD1 and CAT levels to evaluate oxidative stress status in the cytosol of the lung tissues. SOD1 levels decreased in the SOD1^G93A^ rats of group A (*p ≤ 0.05), where they increased in group C (insignificant) and D (**p ≤ 0.001). CAT levels decreased in the mutated rats of groups 0 (*p ≤ 0.05), A (insignificant), and B (insignificant); however, increased in groups C (*p ≤ 0.05) and D (*p ≤ 0.05) (Figure 9).

## Discussion

ALS is the most common motor neuron disease, and the incidence is increasing worldwide every year. Since there is no treatment against ALS, therapeutic approaches are urgently needed to cure people with ALS (Aydemir and Ulusu, 2020b). The lung is one of the most affected organs during ALS progression; however, no studies address possible mechanisms behind the lung impairment in patients with ALS that can be used as a promising targeted therapy approach in the future (Brent et al., 2020). Biomechanical and biochemical properties of the lung have been impaired in several diseases such as Parkinson’s Disease, pulmonary fibrosis, cancer, asthma, emphysema, chronic obstructive pulmonary disease (COPD), and pulmonary arterial hypertension (PAH) disease according to the literature; however, no published data addressing possible mechanisms contributing to the ALS progression (Burgstaller et al., 2017). Therefore, in this study, we aimed to investigate the impact of ALS on the biomechanical changes correlated with oxidative stress metabolism and mutant SOD1^G93A^ protein accumulation in the lung tissue. Since aging directly contributes to tissue dysregulation, such as increased stiffening, we used control animals for each age group to eliminate the effects of aging as described in the section of animal studies (Sicard et al., 2018).

Since increased lung stiffness has been observed in several diseases due to impaired elasticity, altered biochemical and biomechanical homeostasis of the lung tissue (Wells and Hirani, 2008), we first measured lung stiffness to evaluate biomechanical changes during ALS progression by eliminating effects of aging. The stiffness of the biological specimens can be estimated experimentally using tensile testing equipment. The modulus of elasticity can also be used to frame the mechanical behavior of the biological samples using tensile testing equipment. The force divided by the cross-sectional area of the sample is termed the stress, and the displacement divided by the unstressed length of the specimen is termed the strain. The slope in the linear region of the stress versus strain curve is defined as a modulus of elasticity of the test specimen (Maikos et al., 2008). Due to the small size and non-uniform symmetry of the lungs samples, it is challenging to estimate the exact cross-sectional area to calculate elastic modulus. Therefore, in this study, only the stiffness measurements are considered for subsequent analysis and comparison via a custom-built-up stretcher device (De Kegel et al., 2018; Aydın et al., 2019; Sezgin et al., 2019). For the first time in the literature, we showed that lung stiffness increased in the SOD1^G93A^ mutated male rats compared to the SOD1^WT^ rats even at the very early stages of life, indicating impaired tissue function (Figure 2).

**FIGURE 3:**
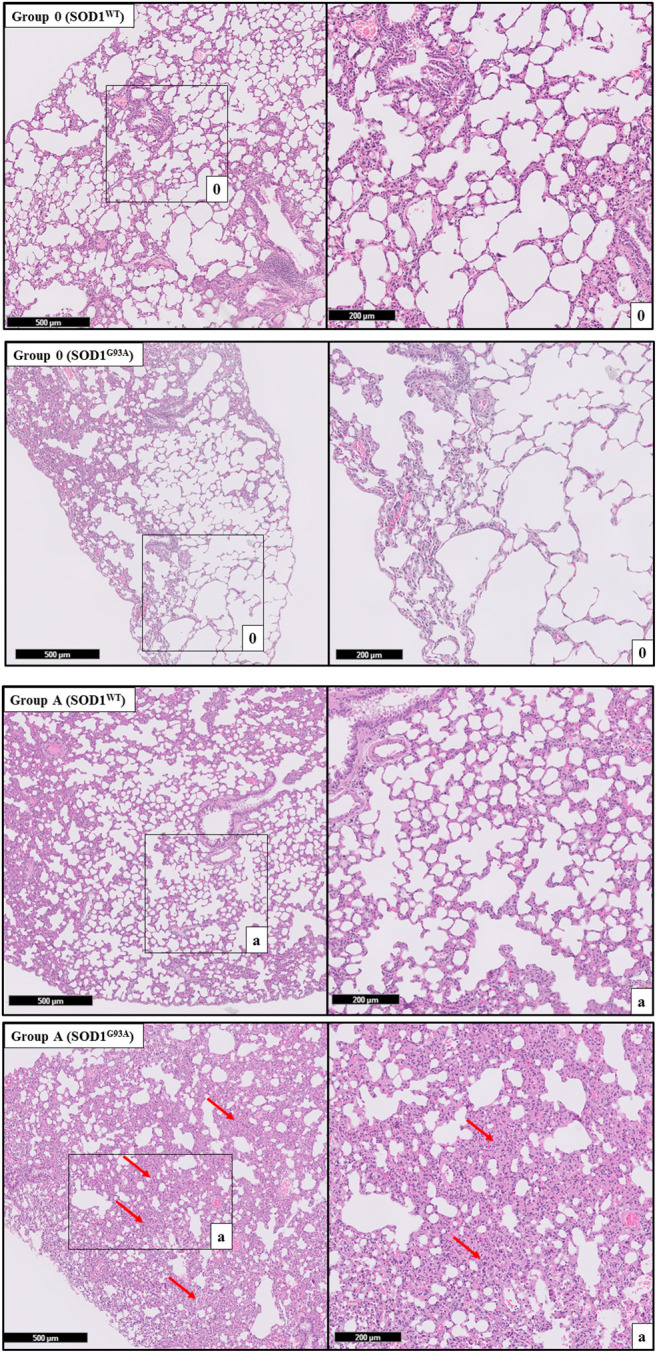
H&E-stained lung tissue sections of lung tissues. Representative images of SOD1^G93A^ and SOD1^WT^ rats of groups 0 and A were taken from each group. Scale lines on the left row indicate 500 μm and the second row 200 μm. Red arrows indicate the area with atelectasis of SOD1^G93A^ rats.

**FIGURE 4:**
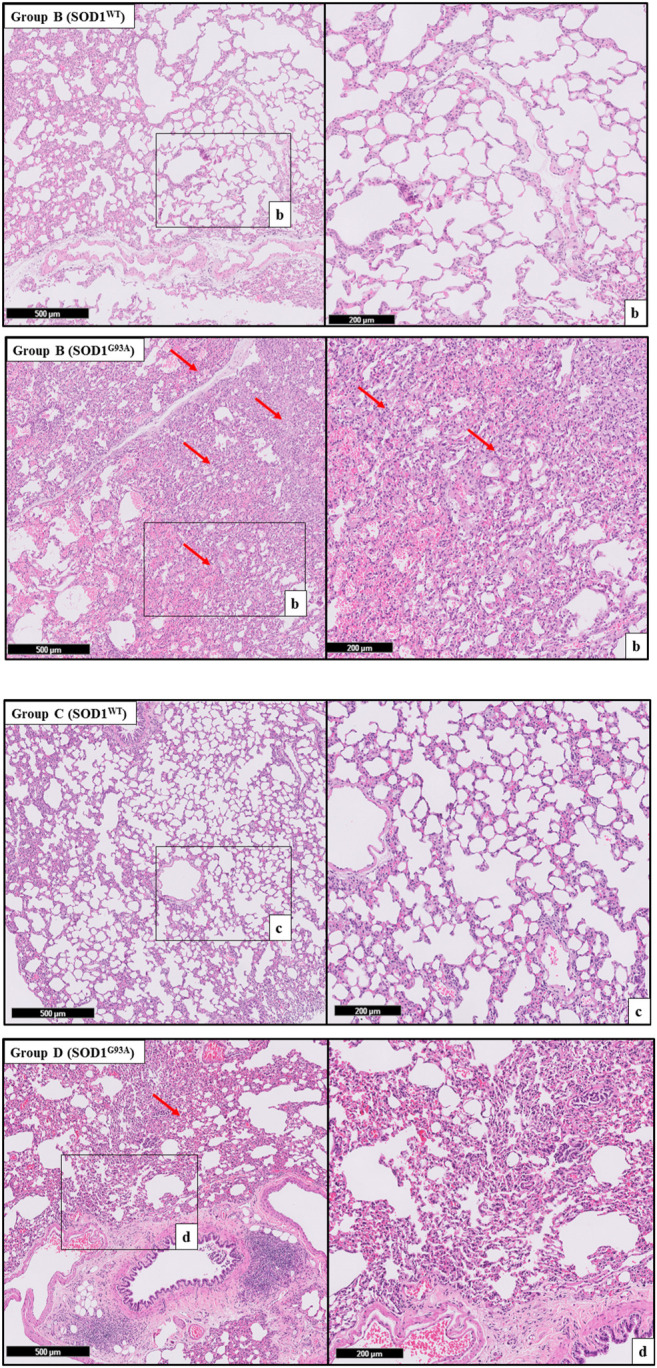
H&E-stained sections of the lung tissue samples belonging to the SOD1^WT^ and SOD1^G93A^ rats of groups B and C. Representative images were taken from each group. Scale lines on the left row indicate 500 μm and the second row 200 μm. Red arrows indicate the area with atelectasis of SOD1^G93A^ rats.

**FIGURE 5:**
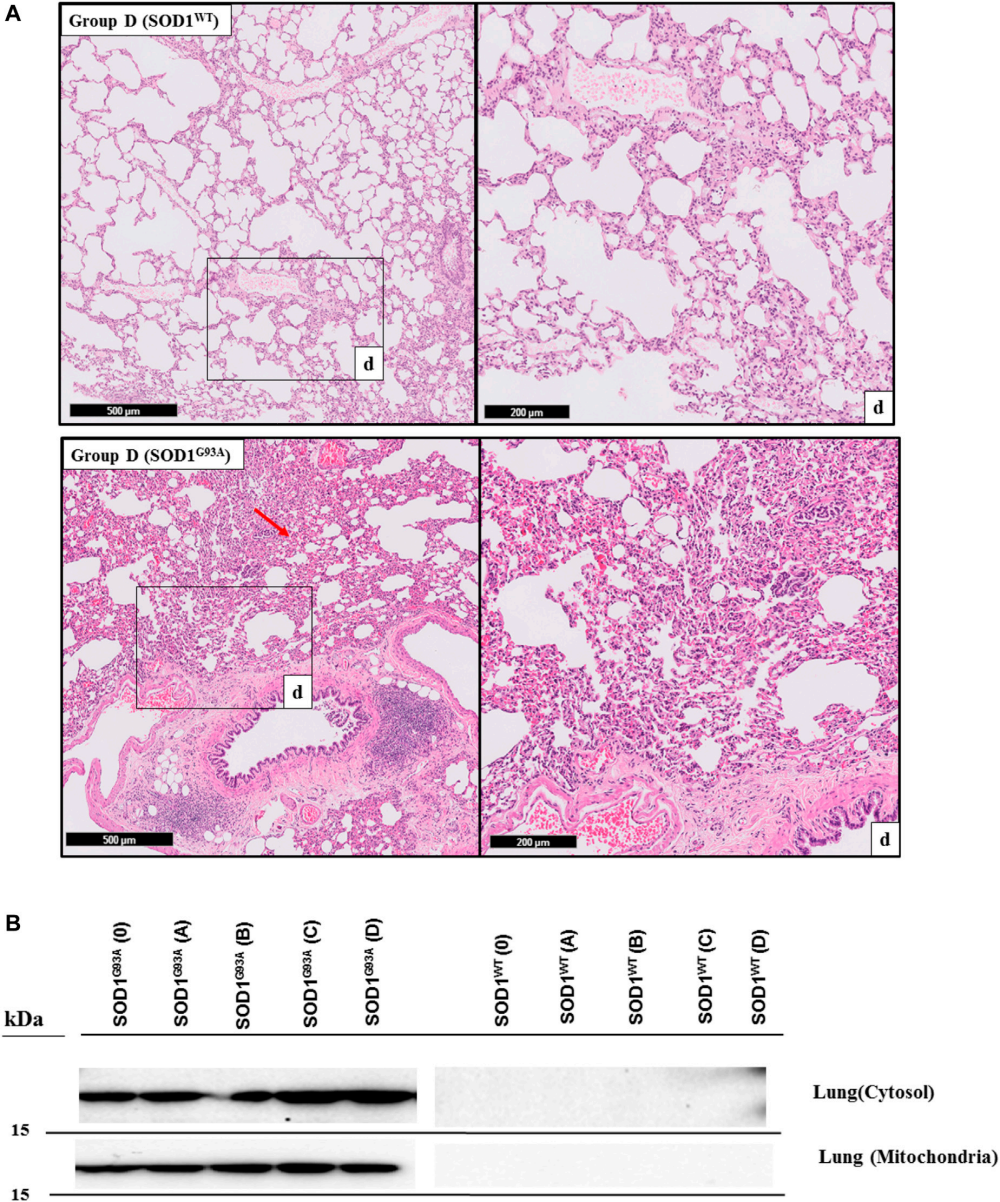
H&E-stained sections of the lung tissue samples belonging to the SOD1^WT^ and SOD1^G93A^ rats of group D. All lung tissues of rats (*n* = 90) were stained and evaluated; among them, representative images were taken from each group. Scale lines on the left row indicate 500 μm and the second row 200 μm. Red arrows indicate the area with atelectasis and emphysema of SOD1^G93A^ rats (A). SOD1^G93A^ protein accumulation was evaluated in the cytosolic and mitochondrial fractions of the lung tissue. Accumulation of human mutant SOD1^G93A^ has been found in all SOD1^G93A^ groups, where SOD1^WT^ did not show any mutant protein in the lung tissue (B).

Increased stiffness causes impairment in lung tissue’s biomechanical properties, which can be evaluated by the histopathological evaluation; therefore, we performed H&E staining for all lung samples to compare mutated and wild-type rats (Cacopardo et al., 2021). Each lung sample was evaluated after H&E staining and compared between groups by a pathologist. Substantial atelectasis (∼30%–60%) and some emphysemas were observed in the lung tissue of mutated rats of all groups except groups 0 according to our data (Figures 3–5). Atelectasis leads to the impairment in the lung elasticity and blood oxygenation, causing inflammation, impairment in the alveolar-capillary barrier, tissue damage, and respiratory function at the end (Zeng et al., 2021). Atelectasis and tissue stiffness started at the early stages of ALS progression, indicating tissue damage even at the pre-symptomatic stages (Figures 3–5). Increased tissue stiffness and histopathological changes indicating tissue dysfunction have been found in the lung samples of SOD1^G93A^ rats compared to SOD1^WT^ rats (Figures 2–5). Elasticity, homeostasis, and stiffness of lung tissue are mainly regulated by ECM regulation. According to the literature, extracellular matrix (ECM) dysregulation, protein aggregation, impaired mineral metabolism, aging, and trace element accumulation are essential mechanisms contributing to lung stiffness (White, 2015). Moreover, ECM regulation is affected by mineral composition, matrix modifying enzymes, oxidative stress, and remodeling processes (Liu et al., 2010; Parameswaran et al., 2011; White, 2015).

**FIGURE 6:**
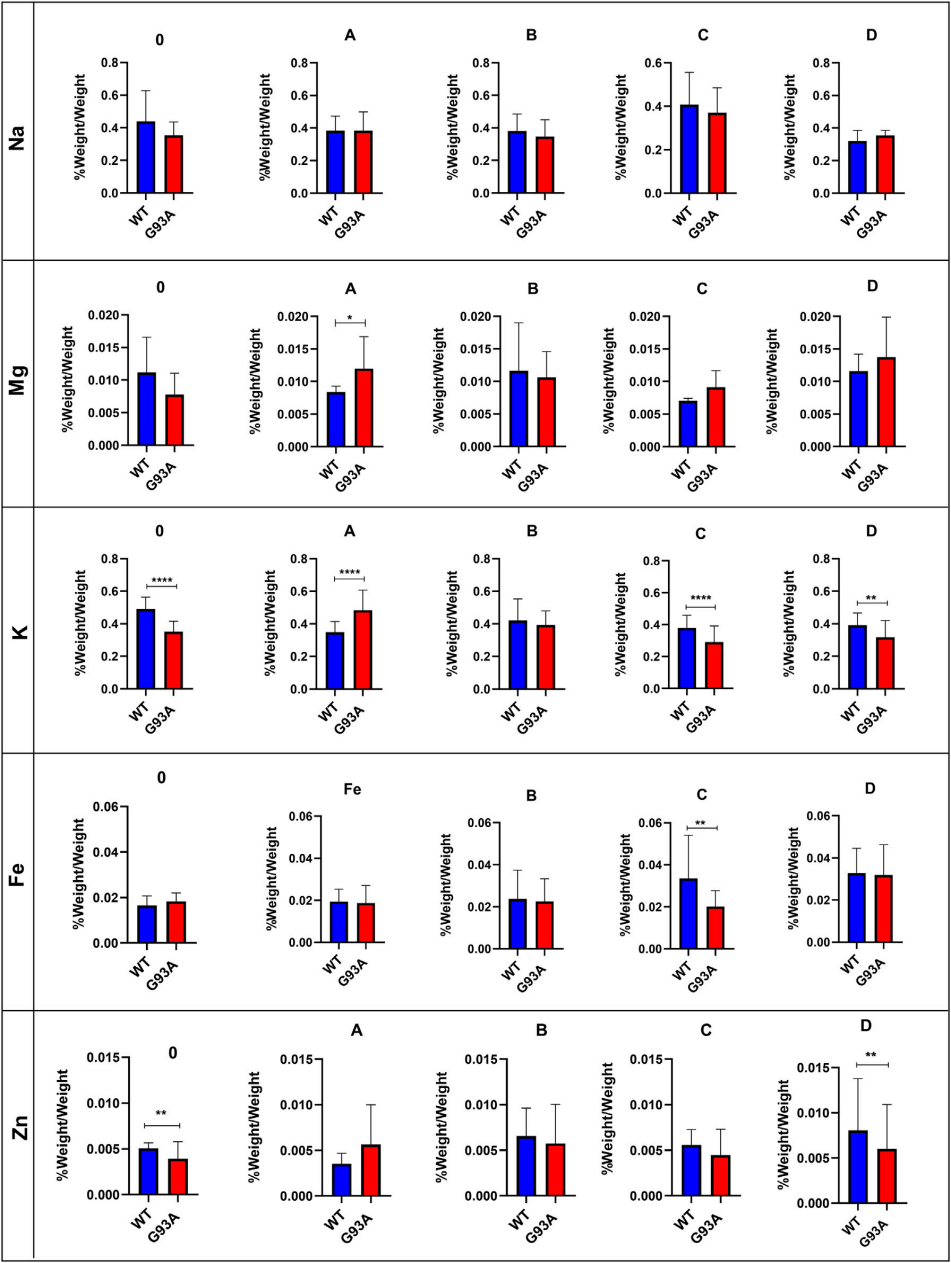
Trace element and mineral levels of SOD1^G93A^ and SOD1^WT^ rats. *p ≤ 0.05, **p ≤ 0.001 and ***p ≤ 0.0001.

Trace element and mineral levels are vital for tissue homeostasis, cellular functions, oxidative stress metabolism, and aging (Aydemir et al., 2020a); thus, we have evaluated Na, Mg, K, Fe, and Zn levels of the lung tissue. Zn levels decreased in the mutated rats of groups B, C, and D compared to the wild-type rats (Figure 6). Zn plays a role in the matrix metalloproteinase (MMP) enzyme activities regulating ECM homeostasis and oxidative stress metabolism as integrating into the structure of the oxidative stress enzymes (Kostov and Halacheva, 2018). On the other hand, Na and K levels fluctuated in the SOD1^G93A^ rats compared to SOD1^WT^ rats indicating impaired electrolyte levels and impaired mineral metabolism in the lung tissue (Figure 6). According to the literature, Na, K-ATPase function has altered the alveolar epithelial barrier leading to tissue damage in the lung (Vadász et al., 2007), and Na^+^ pumps are responsible for the creating Na^+^ gradient to keep the balance of pulmonary liquid balance. Additionally, Na^+^ pumps and balance are involved in the epithelial stretch, vital for breathing and oxygenation (Fisher and Margulies, 2002). K levels significantly decreased in the mutated rats, where Na levels fluctuated in the lung samples of SOD1^G93A^ rats during ALS progression, according to our data (Figure 6). As a result, Zn, K, Mg, and Na levels are impaired in the SOD1^G93A^ rats compared to the SOD1^WT^ rats indicating altered homeostasis in the MMP activity, ECM regulation, oxidative stress metabolism, and Na, K-ATPase function (Figure 6) that contribute to the impaired oxidative stress metabolism and lung function.

Several theories address possible mechanisms behind ALS pathogenesis; among them, protein aggregation has been tightly correlated with dysfunction in the cellular function, oxidative stress metabolism, and tissue damage (Zhao et al., 2010; Hayashi et al., 2016; Kaur et al., 2016). Additionally, impaired Na, K, Zn, and Mg levels can be considered indicators of impaired oxidative stress metabolism (Aydemir et al., 2018). Thus, we hypothesized that tissue damage and dysfunction could result from elevated oxidative stress levels induced by the accumulation of human mutant SOD1^G93A^ protein in both mitochondria and the cytosol of lung tissues (Figure 6) since oxidative stress impairs ECM regulation and tissue homeostasis (Watson et al., 2016). On the other hand, pulmonary endothelial barrier in the lung tissue is affected by elevated levels of oxidative stress resulting in the dysregulation of biomechanical changes of the lung (Haak et al., 2018). During breathing cycle, blood passes through pulmonary vessels and distension occurs in the lung leading to the formation of biomechanical forces such as fluid shear stress, pressure and cyclic stress. Indicated biomechanical changes cause formation of oxidative stress in the lung, thus maintenance of anti-oxidant defense and redox metabolism play vital role to protect lung homeostasis (Zemskov et al., 2019). Oxidative stress is elevated by both ageing and diseases causing to the impaired inflammatory function, pulmonary endothelial barrier function, apoptosis and ECM dysregulation in the lung. On the other hand, oxidative stress is considered is one of the major hallmarks several lung diseases including chronic obstructive pulmonary disease (COPD), acute respiratory distress syndrome (ARDS) and idiopathic pulmonary fibrosis (IPF). Since we have proven that mutant SOD1G93A protein accumulated and histopathological changes have arisen in the lung tissue, oxidative stress parameters have evaluated as major cause of the impairment in the lung function during ALS progression (Hecker, 2018).

Oxidative stress is produced in all aerobic cells; however, neurons are more susceptible to oxidative stress. Antioxidant protection of the organism is maintained by three enzymes, including the SOD enzyme family, catalase, and GSH-dependent enzymes such as glutathione reductase (GR), glutathione-s transferase (GST), and glutathione peroxidases (GPx). The redox metabolism in the organism is regulated by reduced glutathione (GSH), oxidized glutathione (GSSG), GSSG/GSH ratio, catalase (CAT), superoxide dismutase family (SOD), NAD+/NADH, NADP+/NADPH, glucose-6 phosphate dehydrogenase (G6PD), 6-phosphogluconate dehydrogenase (6-PGD), GR, GPx and GST. NADPH is a reducing agent and plays a vital role in the detoxifying processes by eliminating oxidative radicals and peroxides (Ozcan et al., 2021). Additionally, it involves various metabolic processes, including cell proliferation, survival and senescence, energy metabolism, mitochondrial functions, calcium homeostasis, antioxidant/generation of oxidative stress, gene expression, fatty acid, and steroid synthesis. On the other hand, NADPH is required for the amino acid synthesis involved in the cytochrome P450 systems enabling detoxification of drugs, redox balance, immunological homeostasis, aging, and cell death. Decreased levels of G6PD and 6-PGD are the indicators of impaired glucose and energy metabolisms in both cytosol and mitochondria as well (Aydemir et al., 2019a; Aydemir and Ulusu, 2020a; Aydemir et al., 2020b).

**FIGURE 7:**
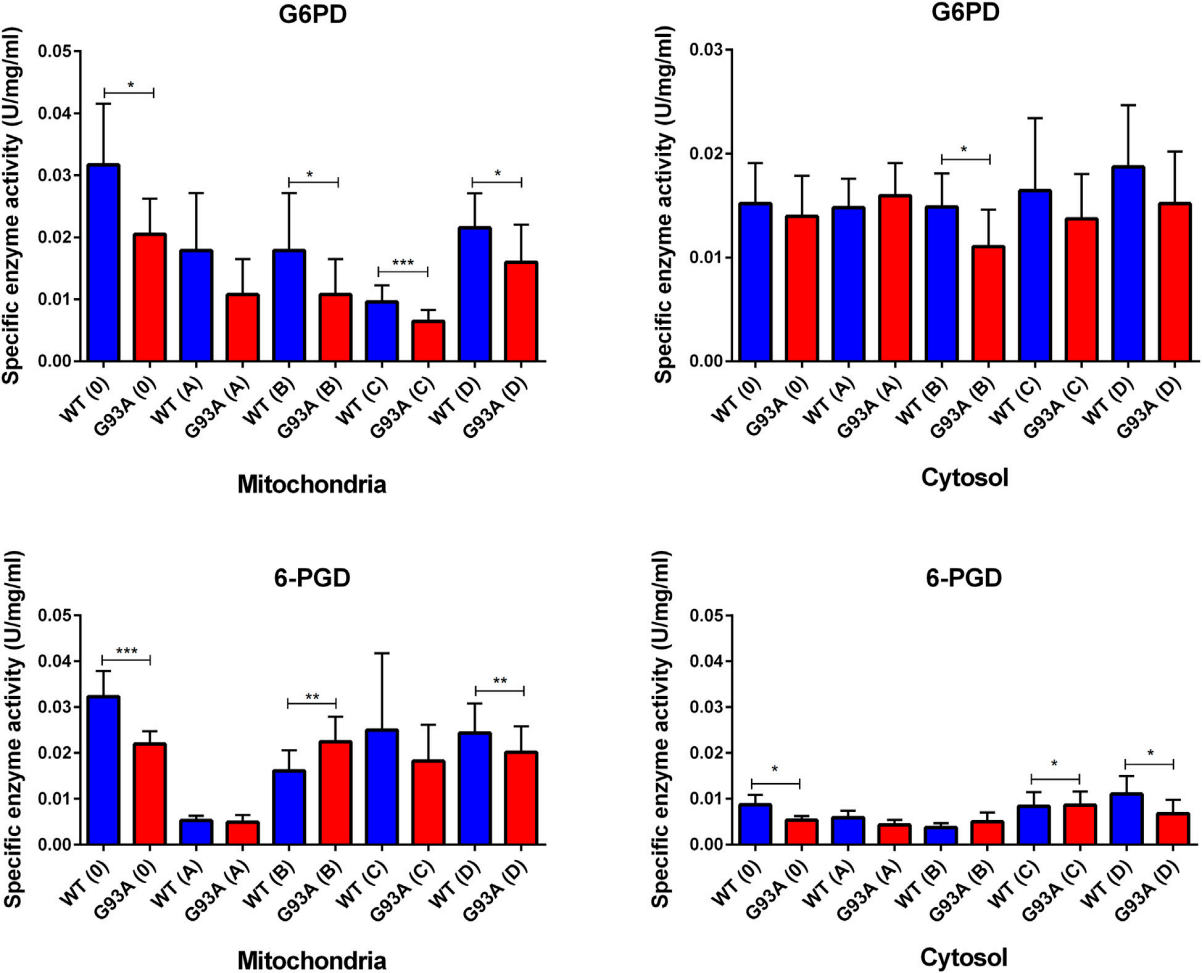
G6PD and 6-PGD enzymes belonging to the PPP were evaluated in both cytosol and mitochondria of lung tissues of SOD1^WT^ (WT) and SOD1^G93A^ (G93A) rats. Data were given as mean ± SD of n = 8 animals for each group. Notes: *p ≤ 0.05, **p ≤ 0.001 and ***p ≤ 0.0001.

**FIGURE 8:**
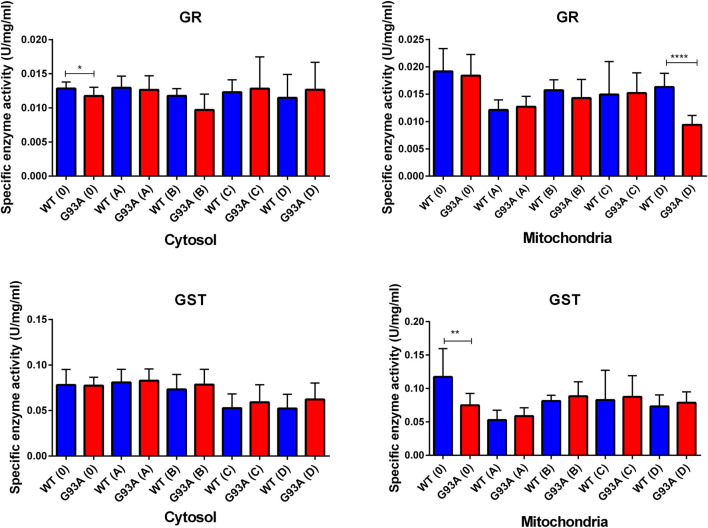
GR and GST enzymes were evaluated in both cytosol and mitochondria of lung tissues of SOD1^WT^ (WT) and SOD1^G93A^ (G93A) rats. Data were given as mean ± SD of n = 8 animals for each group. Notes: *p ≤ 0.05, **p ≤ 0.001 and ***p ≤ 0.0001.

**FIGURE 9:**
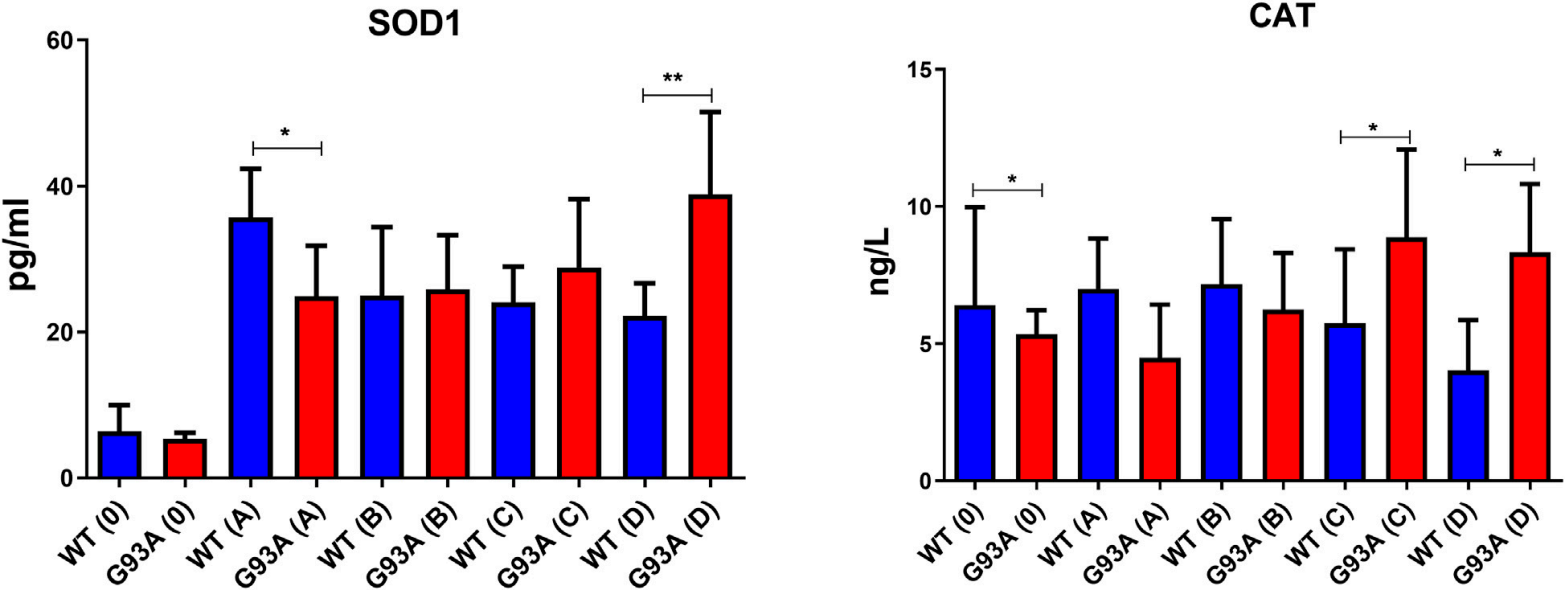
SOD1 and CAT levels in the cytosol of lung tissues belonging to both SOD1^WT^ (WT) and SOD1^G93A^ (G93A) samples were evaluated via ELISA assay. Data were given as mean ± SD of n = 8 animals for each group. Notes: *p ≤ 0.05, **p ≤ 0.001 and ***p ≤ 0.000.1.

NADPH enables the reduction of GSSG to its reduced form GSH, which is a major scavenger of ROS and required for many reduction systems. G6PD and 6-PGD enzymes reduce NADP into NADPH, so they remain NADPH pool in the cells and keep redox balance. Imbalance or malfunction in the redox pathways of antioxidant enzymes causes impairment in the antioxidant metabolism leading to elevated levels of oxidative stress (Aydemir et al., 2018; Aydemir et al., 2019a; Aydemir and Ulusu, 2020a; Aydemir and Ulusu, 2020b; Aydemir et al., 2021). G6PD and 6-PGD levels decreased in SOD1^G93A^ mutated rats in both cytosol and mitochondria (Figure 7), which may lead to the reduced antioxidant status and elevated levels of oxidative stress. Additionally, GR and GST levels increased in the SOD1^G93A^ rats compared to the SOD1^WT^ rats indicating elevated levels of oxidative stress (Figure 8)). We further evaluated CAT and SOD1 levels to prove high levels of oxidative stress and showed that CAT and SOD1 levels significantly increased in the SOD1^G93A^ rats compared to the SOD1^WT^ rats (Figure 9). As a result, we found that oxidative stress metabolism is impaired in the mutated rats because of the accumulation of mutant SOD1G93A protein that can be considered a possible mechanism behind increased lung stiffness and impaired tissue function. In the future, possible targeted therapy approaches can be tried to improve lung function to prolong survival and improve the life quality of ALS patients.

## Conclusion

Decreased antioxidant status, elevated levels of oxidative stress, and mutant SOD1^G93A^ protein accumulation have been found in the lung samples of SOD1^G93A^ mutated rats even at the earlier stages of life first time in the literature, which can be possible causative of increased stiffness and impaired morphology of lung tissue. All these changes can result from the accumulation of SOD1^G93A^ rats in mitochondria and cytosol, causing impaired energy and oxidative stress metabolism in the lung. Since indicated alterations have started at the early stages of ALS progression, even in the pre-symptomatic stages, possible therapeutic approaches can be used to treat ALS or improve the life quality of patients with ALS.

## Data Availability

The original contributions presented in the study are included in the article/Supplementary Material, further inquiries can be directed to the corresponding author.
